# Applications of the *BLEND* Software to Crystallographic Data from Membrane Proteins

**DOI:** 10.1007/978-3-319-35072-1_9

**Published:** 2016-04-28

**Authors:** Pierre Aller, Tian Geng, Gwyndaf Evans, James Foadi

**Affiliations:** 1 Diamond Light Source Ltd, Harwell Science and Innovation Campus, OX11 0DE Didcot, UK; 2 Membrane Protein Laboratory, Diamond Light Source Ltd, Harwell Science and Innovation Campus, OX11 0DE Didcot, UK

**Keywords:** Blend, Multiple crystals, Membrane proteins, Data scaling, Data merging, Isomorphism, Cluster analysis, Radiation damage

## Abstract

X-ray diffraction from crystals of membrane proteins very often yields incomplete datasets due to, among other things, severe radiation damage. Multiple crystals are thus required to form complete datasets, provided the crystals themselves are isomorphous. Selection and combination of data from multiple crystals is a difficult and tedious task that can be facilitated by purpose-built software. *BLEND*, in the CCP4 suite of programs for macromolecular crystallography (MX), has been created exactly for this reason. In this chapter the program is described and its workings illustrated by means of data from two membrane proteins.

## Introduction

The correct interpretation of X-ray diffraction patterns for macromolecules is nowadays routine. A few computer programs have reached the status of mature and robust applications capable of suggesting the most likely symmetry groups and cell parameters for the protein under study as well as carry out data integration (Otwinowski and Minor 1997; Leslie and Powell 2007; Kabsch 2010; DIALS^2015^). The subsequent scaling of integrated intensities and the extraction of structure factors, to be later used in the solution process, does not represent a problem if the diffraction data collected are complete and if unique intensities have sufficient multiplicity (French and Wilson 1978; Kabsch 2010; Evans and Murshudov 2013). Straightforward application of these packages, though, is typically limited to soluble proteins for which good-size crystals can be easily obtained and for which Radiation damage does not restrict the collection rotation range. However, X-ray data from other classes of macromolecules, like membrane proteins or macromolecular complexes, are less easy to integrate and scale. Complicating factors such high-resolution diffraction spot weakness, due to the increased solvent content and lattice disorder, severe anisotropy, due to limitations in crystal packing along all spatial directions, and incomplete reciprocal space covering, due to crystal decay during X-ray exposure are the most common. The traditional way to deal with such difficulties is to spend a considerable amount of time optimising Crystallisation conditions aiming at the formation of relatively large and homogeneous crystals, capable of scattering at high resolution and to withstand Radiation damage for longer collection times. Progress at third generation synchrotrons has recently made it feasible to collect data from small crystals, even at room temperature, hence opening a new mode of collecting data from difficult crystals (Axford et al. 2012, 2015; Aller et al. 2015). In the case of membrane proteins, one tries to enhance spots signal over the scattering background by increasing X-ray beam flux and/or exposure time. This is especially made possible by the adoption of new high-frame-rate pixel-array detectors (Broennimann et al. 2006). The images produced will be easier to index and integrate, but the collection range is severely limited, as the crystal’s diffracting power will rapidly decrease because of Radiation damage. As a result, small wedges of reciprocal space are sampled, leading to inaccurate estimates of cell parameters and integrated intensities. If several isomorphous crystals are available, data accuracy can be improved by averaging contributions from different data collections on individual crystals. Close isomorphism does not, in general, occur and crystals will be formed with a varying degree of non-isomorphism. If cell parameters between two crystals do not change considerably, such crystals can be effectively considered as isomorphous. Therefore, data merging from the relative intensities can potentially provide a dataset with increased completeness. An important aspect in the new approach to collect data from membrane proteins crystals is, therefore, the availability of software to analyse data from Multiple crystals and single out isomorphous groups in order to generate complete datasets.

In this chapter the program BLEND (Foadi etal. 2013), part of the CCP4 suite of programs (Winn et al. 2011), will be introduced and explained. The chapter is specifically centred on applications of the program to assemble complete data for membrane proteins.

## Main Ideas and Structure of the BLEND Program

BLEND has been developed to help give guidance to users on merging data from the most isomorphous crystals using Cluster analysis. The statistical descriptors for Cluster analysis used in BLEND are the unit cell parameters (a, b, c, α, β, γ). Before running *BLEND* all single datasets from the different crystals must be integrated with either *XDS* (Kabsch 2010), MOSFLM (Leslie and Powell 2007) or DIALS (DIALS 2015). *BLEND* can read HKL files from *XDS* and unmerged MTZ files from *MOSFLM* or *DIALS. BLEND* can, at present, be executed in five different modes: *dendrogram-only*, *analysis*, *synthesis*, *combination*, and *graphics* modes.

Typically, the *analysis* mode is run first. The program reads integrated reflection files and runs Cluster analysis on cell parameters by default. The analysis mode will generate a tree or a dendrogram to display which datasets are isomorphous. The user must be aware that dendrograms will be generated even in case of close isomorphism. Hence, caution is required when scrutinizing these dendrograms. The Linear Cell Variation (LCV), or its absolute equivalent (aLCV) values, calculated for all clusters (see BLEND documentation at http://www.ccp4.ac.uk/html/blend.html) might help in the interpretation of the tree. Indeed the LVC indicator shows the largest difference between two datasets; some non-isomorphism between datasets is generally to be expected with LCV values greater than 2 %. It often happens that the *analysis* mode shows strong outliers (high LCV) making the interpretation of the dendrogram difficult. Removing these outliers and re-running *BLEND* in *analysis* mode generally provides a clearer tree to interpret.

The second step is the execution in *synthesis* mode. During this step the user will decide which datasets to merge together based on the dendrogram’s results. *POINTLESS* (Evans 2006) and AIMLESS (Evans and Murshudov 2013) will be run automatically on each selected cluster, and the statistics after scaling (R_meas_, R_pim_, and Completeness) are also displayed. Users can choose which datasets/clusters are the best fit for further work, based on the statistics provided. For example a cluster with reasonable completeness and lowest R_meas_/R_pim_ can often be the best option. Some other times, weak merging statistics can be accommodated in favour of higher resolutions. All files generated by the *synthesis* mode are stored in a directory named “merged_files”. These are reflection files in MTZ format before and after scaling, and the corresponding logs from *POINTLESS* and *AIMLESS* for each cluster can be found. When fine-tuned scaling options are needed, users are advised to execute *AIMLESS* jobs starting from the unscaled reflection files obtained from previous executions of BLEND.

The *combination* mode can be run any time after the *analysis* mode or the *synthesis* mode. This mode adds flexibility to the program. It allows the user to merge any datasets/clusters of choice. For instance, here the user might choose to merge together datasets shown to belong to distant clusters during the previous run *in synthesis* mode. BLEND merges these groups of datasets running *POINTLESS* and AIMLESS, even though the specific group was not contemplated during *synthesis* mode because no clusters in the dendrogram contained those specific datasets.

More details and explanations of the various modes and options available in BLEND can be found in the following sections.

## Membrane Proteins Used in This Chapter

Data from crystals of two different membrane proteins have been included in this chapter to help demonstrate BLEND workings. The first is Hemophilus Influenza TehA (Chen et al. 2010; Axford et al. 2015) and the second is the Human Histamine Receptor H1R (Shimamura et al. 2011) in complex with a second-generation anti-histamine drug (Heifetz et al. 2015). These proteins have been chosen because data were readily available and because *BLEND* is applied differently to the two types of datasets, facilitating a broad display of its functionality. The data for TehA consist of 67 small reciprocal space wedges collected from 56 different crystals *in situ* at room temperature and the structure was solved at 2.3 Å resolution (Axford et al. 2015). The data for the H1R consist of 18 partially complete or complete datasets from 18 different crystals that were collected at 100 K. This structure was solved at 3 Å resolution. Data for both structures were collected at the microfocus beamline I24 of the Diamond Light Source synchrotron. All data were included in a directory referred here for convenience as $BTEST.

## Dendrogram-Only Mode (−aDO): Quick Clustering and Data Bookkeeping

Cluster analysis on groups of cell parameters helps to form first impressions on collected data and to organise them for later processing. The starting input for BLEND is integrated data that have not yet been scaled and merged. As mentioned earlier, these can either be MTZ unmerged binary files (produced by the integration programs MOSFLM*and*DIALS) or HKL ASCII files produced by *XDS*. To further clarify this point, *XDS* yields two types of HKL files. The first is normally called “INTEGRATE.HKL” and it includes integrated and unscaled intensities for all non-unique reflections. The correct space group is normally assigned in “XDS_ASCII.HKL”, but not within “INTEGRATE.HKL” where, in general, intensities are assumed to describe a structure without symmetry (P1). Scaling and estimation of space group is done by the *XDS* CORRECT module. Scaled (but unmerged) intensities with final space group information are included in “XDS_ASCII.HKL”. Although *XDS* files of the “XDS_ASCII.HKL” type can be fed into *BLEND* without any apparent warning, this is not recommended because the program eventually tries to scale all data with AIMLESS. With the “XDS_ASCII.HKL” files this would mean to scale once more intensities that had already been scaled. Thus the advice is to use intensities in the “INTEGRATE.HKL” file types. All input data can be included in the same directory, or spread across a number of different directories. The command line to execute *BLEND* will vary in the two cases.

### All Input Files in a Same Directory

#### TehA

Assuming that the TehA data in MTZ format are all included in a single directory called “$BTEST/TehA”, a quick clustering is obtained by running BLEND in *dendrogram-only* mode (-*aDO*) using the following command line:

blend -aDO $BTEST/TehA

**Fig. 9.1:**
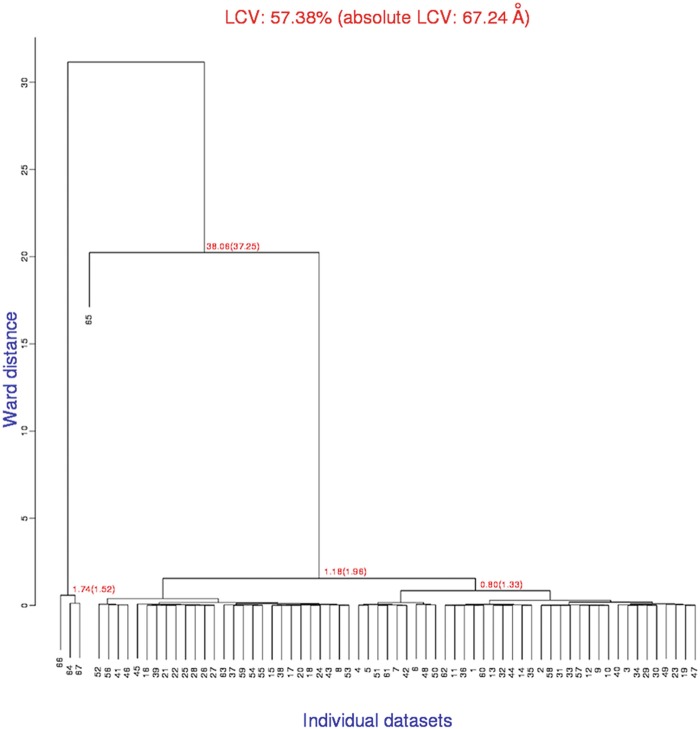
Dendrogram produced by BLEND for the TehA test data. The *red numbers* are LCV values with aLCV values *within brackets*. Four of the 67 datasets analysed have unusually large LCV values. This, typically, indicates incorrect indexing, normally caused by crystal quality or data collection specific issues

The program starts and halts immediately after, waiting for input keywords. Pressing the Enter key is equivalent to accepting default values for all keyworded procedures and parameters. After a few seconds the program ends successfully. Among all files produced by BLEND the most important to look at is the dendrogram produced. For the present protein case, this is shown in Fig. 9.1.

The dendrogram represents proximity between various crystals in the whole group of data under investigation. The red numbers are annotations for the top five clusters of the linear cell variation (LCV) and the absolute linear cell variation (aLCV, within brackets). They provide prompt information on crystals isomorphism. High LCV and aLCV values indicate non-isomorphism among crystals in the specific cluster. In Fig. 9.1 most datasets show LCV equal to 1.18 %; this means that the largest difference in size among all crystals in that cluster amounts to 1.18 % of crystals size. A few crystals, though, make the LCV increase to 38.06 % and 57.38 %. This, in general, indicates that the crystals are entirely foreign to the structure under investigation or, as the case presented here, the crystal datasets have been indexed incorrectly by the integration program. At this point one should analyse carefully the integration stage for these “dendrogram outliers” and either index them correctly, or discard them if indexing remains difficult to achieve. It turns out that datasets 64, 65, 66 and 67 had all been collected with the X-ray beam placed at the interface between contiguous crystals, giving rise to overlapping reciprocal lattices, not neatly interpretable as belonging to a specific space group. The choice in this specific case was to discard the four datasets with the remaining 63 datasets appearing to be substantially isomorphous (LCV = 1.18 %). These were divided into two main clusters with cell parameters showing smaller differences (LCV = 0.68 % for the left branch and LCV = 0.80 % for the right branch).

#### H1R

For this crystal structure, 18 datasets, many of them fairly complete, have been collected from 18 different cryo-cooled crystals in multiple-collection episodes. As none of the individual datasets was considered to be satisfactory in terms of data quality or resolution, it was, decided to carry out multiple-crystals analysis with the hope of improving resolution and obtaining an interpretable electron density.

A first quick analysis was performed with the following command line:

blend -aDO $BTEST/H1R

**Fig. 9.2:**
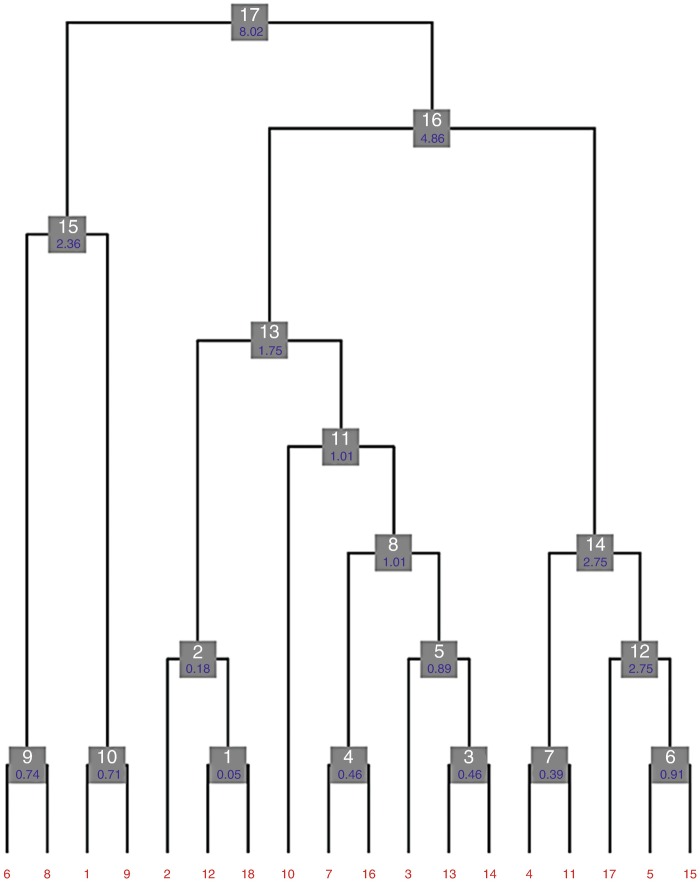
Dendrogram with annotated aLCV values for H1R datasets. In this dendrogram the various nodes, here represented as *grey boxes*, are not displayed at their usual cluster heights but, rather, at their merging level. *Boxes* at a same level describe clusters with equal numbers of datasets

The dendrogram produced is shown in Fig. 9.2. The various groups in the tree are not shown at their usual cluster height, but, rather, at the merging level, where nodes at a same level correspond to clusters with equal number of datasets. For example, clusters 9, 10, 1, 4, 3, 7, 6 are plotted at the lowest level because they correspond to the first merging, when two individual datasets are joined into one cluster; clusters 2, 5, 12 are plotted at the second level because they correspond to the second merging (like cluster 6 and dataset 17 joining into cluster 12), with all three clusters being formed by three datasets. This type of annotated dendrogram is very useful as it gives an overview of crystals isomorphism at the unit cell level. In this case, for example, it is clear that the three top clusters 13, 14 and 15 have an acceptable level of isomorphism at 3 Å resolution, but their union into larger clusters is bound to introduce some considerable structural differences, resulting in map artefacts or distortions.

### Input Files in Different Directories

In the H1R case, data have been collected in multiple instances at different times. Such data had to be copied into a same directory in order to run BLEND as described in the previous section. Alternatively, data can be left in the original location and an ASCII file can be written to list their relative computer locations (paths). This file becomes the new input for *BLEND*.

#### TehA

As previously shown, datasets 64, 65, 66 and 67 are outliers. A new ASCII file can be prepared in which these datasets are excluded. This is easily done if files are available from a previous run. More specifically, the mtz_names.dat file that was produced by the previous run in *dendrogram-only* mode and contains paths to all 67 datasets can be copied, given a new name, say “original_TehA.dat” and edited in order to remove the unwanted datasets. BLEND can be subsequently executed with the following command line

blend -aDO original_TehA.dat

pressing the Enter key as required (see Sect. 9.4.1.1)

#### H1R

The mtz_names.dat file created by the previous run in *dendrogram-only* mode (see Sect. 9.4.1.2) can be copied to a new file, let us name it “original_H1R.dat”, in which some of the least homogeneous datasets are excluded. The user then can execute BLEND in *dendrogram-only* mode in the following way:

blend -aDO original_H1R.dat

The result will be a tree slightly changed. The procedure can be repeated again with the exclusion of the least isomorphous datasets, until convergence to low values of aLCV are reached.

Later in this chapter we will follow a different approach by calculating merged datasets out of all those clusters having aLCV values smaller than a predefined value (see Sect. 9.6).

## Analysis Mode (–a): Clustering, Radiation damage and Resolution Estimate

Complete analysis of input data is achieved by running BLEND in *analysis* mode (−*a*). This is also a necessary step for later executions in *synthesis* and *combination* modes. The starting point for this section will be either the original_TehA.dat or original_H1R.dat files that are modified copies of the mtz_names.dat files obtained during previous runs in *dendrogram-only* mode.

### TehA

Only 63 datasets are listed in the original_TehA.dat file and the command line for its execution in *analysis* mode is

blend -a original_TehA.dat

**Fig. 9.3:**
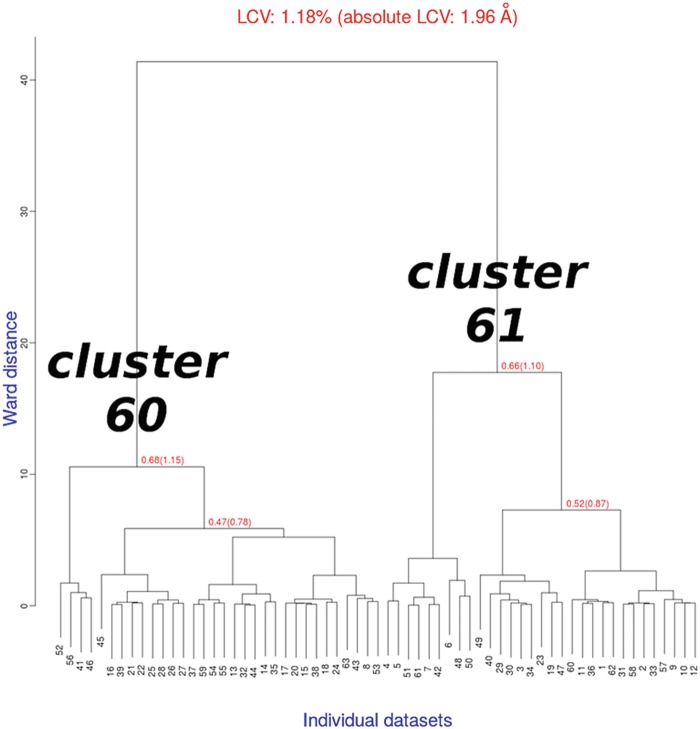
Dendrogram produced by BLEND (*analysis* mode) for TehA test data in which datasets 64, 65, 66 and 67 have been removed

The dendrogram derived from this run is shown in Fig. 9.3. Essentially, the dendrogram produced here is a subsection of the dendrogram shown in Fig. 9.1. Differently from the execution in *dendrogram-only* mode (Sect. 9.4), two additional and important tasks were carried out this time; (1) Radiation damage analysis and (2) estimate of resolution. These are crude procedures with the purpose of filtering out potentially noisy data.

In the Radiation damage*procedure*, the average intensity in each resolution shell is analysed with respect to image number. For each resolution shell, the intensity is expected to decrease with increasing image number in particular for crystals affected by Radiation damage. The observed intensity “decay” is more rapid at high resolutions. Dependencies on image number and resolution are statistically modelled as a linear exponential whose parameters are found using least squares. If the decay parameter is found to be increasing significantly with resolution, then the dataset in study is flagged as being affected by Radiation damage. Knowledge of the modelled parameters can be subsequently used to estimate from which image the intensity severely drops, on average, to a predefined fraction (keyword RADFRAC). All the dataset images after that particular image will be automatically discarded from all the following analyses, unless the user decides otherwise. More details on this procedure can be found in Axford et al. (2015) where is also explained the rigorous validity of the method especially for cases where crystals are small and totally bathed in the X-ray beam (so that its exposed volume does not change during rotation), and the rotation range is small to avoid sizable fluctuations in the primary X-ray beam. All these conditions are applicable to the case study in this section (TehA) where crystals were small and matching the beam size. This procedure is also valid for longer rotation sweeps, provided that the beam is stable, the crystal is not too large, and the exposed volume does not change considerably during rotation.

*Averaging intensities in resolution shells* (this time including all images) are computed to estimate resolution cutoff. Averages are also computed for intensity errors. Ratios of the calculated averages form the starting points for a 10° polynomial interpolation. The resolution at which the polynomial falls below 1.5 is, by default, taken as the suggested highest resolution for the dataset under investigation. The numerical choice of 1.5 is based on tests on several data and is in general a conservative choice. It is important to remember that all the averages, at this stage, are calculated with unscaled data, and scaling in BLEND happens later, when multiple datasets are joined together using AIMLESS. The “Mean((I)/sd(I))” quantity used in *AIMLESS* to judge data quality at various resolution ranges was mostly found to be better than 2 for the greatest majority of tests in *BLEND*. The 1.5 value can, in any case, be changed through keyword ISIGI by the user.

The results from the two procedures described above produced by BLEND is found in a file named

FINAL_list_of_files.dat

The ASCII file is divided into six columns and for each dataset includes the following information:

1. Path to MTZ integrated data
2. Dataset serial number (same as the one used in the dendrogram)
3. Number of last accepted image, as suggested by the Radiation damage procedure
4. Number of first image
5. Number of last image
6. Applied resolution cutoff

The first 12 lines of the FINAL_list_of_files.dat file for the TehA data are shown in Table 9.1.

**Table 9.1:** The first 12 lines of file FINAL_list_of_files.dat produced by BLEND ran in *analysis* mode for the TehA datasets

File name	Dataset serial number	Cutoff image	First image	Last image	Suggested resolution
integrate05.mtz	1	17	1	30	2.614
integrate06.mtz	2	19	1	30	2.146
integrate07.mtz	3	17	1	30	2.105
integrate08.mtz	4	30	1	30	3.230
integrate09.mtz	5	30	1	30	3.450
integrate10.mtz	6	30	1	30	3.855
integrate11.mtz	7	30	1	30	3.290
integrate12.mtz	8	19	1	30	2.089
integrate12_2.mtz	9	21	1	40	2.132
integrate13.mtz	10	19	1	30	2.597
integrate14.mtz	11	18	1	30	2.098
integrate15.mtz	12	18	1	30	2.552

Cutoff images and suggested resolutions are calculated using the procedures described in the main text of Sect. 9.5

Both suggested cutoffs, as estimated by the Radiation damage and by the *resolution* procedures can be modified by the user, either acting on keywords RADFRAC and ISIGI or during the *synthesis and combination* modes, using specific keywords for *POINTLESS* and AIMLESS. As observed in Table 9.1, images cutoff have not been suggested for some of the datasets (datasets 4, 5, 6, 7). It is most probable that Radiation damage has also occurred for these crystals, but either it was not too severe, or the conditions for the algorithm to work were not valid, resulting in the procedure not being applied to the specific dataset.

### H1R

Here, BLEND was executed in *analysis* mode using the file prepared in Sect. 9.4 through the command line below

blend -a original_H1R.dat

During data collection, most of the rotation angle ranges were relatively wide and the crystals not small. Therefore, the procedure to determine radiation damaged parts of data collection was expected not to work well. For this run the values of the FINAL_list_of_files.dat file are listed in Table 9.2.

**Table 9.2:** Values extracted from the FINAL_list_of_files.dat file for the H1R datasets

File name	Dataset serial number	Cutoff image	First image	Last image	Suggested resolution
dataset_003.mtz	1	300	2	300	3.087
dataset_007.mtz	2	107	3	107	2.835
dataset_008.mtz	3	26	1	40	3.849
dataset_009.mtz	4	40	1	40	2.877
dataset_016.mtz	5	100	1	100	3.014
dataset_017.mtz	6	100	1	100	2.894
dataset_019.mtz	7	79	1	79	3.047
dataset_020.mtz	8	100	1	100	3.101
dataset_021.mtz	9	100	1	100	3.135
dataset_023.mtz	10	25	1	40	4.238
dataset_024.mtz	11	35	1	35	2.861
dataset_026.mtz	12	450	1	450	2.588
dataset_027.mtz	13	450	1	450	2.593
dataset_028.mtz	14	422	1	422	2.621
dataset_029.mtz	15	449	1	449	2.546
dataset_030.mtz	16	450	1	450	2.478
dataset_033.mtz	17	224	1	224	2.600
dataset_035.mtz	18	277	1	277	2.565

As observed in Table 9.2, most of the datasets did not present suggested cutoffs. However, a chance to prune damaged parts of the collection for each dataset is given after scaling, using the “pruning” variant of the BLEND combination mode (see Sect. 9.7).

## Synthesis Mode (−s): Obtaining Complete Data

### TehA

The dendrogram in Fig. 9.3 resulted from the *analysis* mode execution has two branches. The aLCV is nearly 2 Å for the two main branches while for each of the secondary branches is just above 1 Å. This occurrence points to a slight non-isomorphism between the two related groups of datasets. Validation of isomorphism in BLEND is achieved with its execution in *synthesis* mode. Details of the various syntax expressions for this mode are included in the official documentation. The aim of the *synthesis* mode execution is to obtain scaled and merged datasets for each cluster of the dendrogram. The top cluster, obtained by merging all datasets together, has height 41.395 (as seen in the file CLUSTERS.txt, produced by *BLEND* earlier in *analysis* mode); thus any number higher than this value will cause *BLEND* to scale and merge datasets for all 62 nodes of the tree. The command line is as follows (the input value “42” has been chosen arbitrarily as a numeric value higher than height 41.395)

blend -s 42 < bkeys.dat

where the ASCII file bkeys.dat includes all keywords needed. As this is the first job in *synthesis* mode, data resolution is still not known (this is normally estimated after scaling). It is, therefore, worth imposing a somewhat ambitious value, selecting the highest resolution among all datasets, as suggested by the run in *analysis* mode, and analyse data after scaling to assess more realistic values. In this specific case study, BLEND suggests a resolution of 2.076 for dataset 42 (the highest among all datasets), thus the keywords file bkeys.dat contains the following line:

RESOLUTION HIGH 2.076

All files produced after execution in *synthesis* mode are grouped in the directory “merged_files”. The first lines of the *summary of overall statistics* listed in file MERGING_STATISTICS.info, regarding to the TehA *synthesis* mode run are shown in Table 9.3.

**Table 9.3:** Statistics after execution of BLEND in synthesis mode for the most complete merged datasets of TehA at 2.08 Å resolution

Cluster number	R_meas_	R_pim_	Completeness	Multi-plicity	Resolution CC_1/2_	Resolution Mn(I/sd)	Resolution Max
62	1.884	0.739	90.60	8.90	2.53	2.15	2.08
60	6.518	3.283	88.10	4.60	3.45	2.16	2.08
58	0.205	0.089	82.90	4.20	2.38	2.15	2.08
57	0.106	0.053	80.10	3.00	2.08	2.16	2.08
61	0.550	0.221	69.80	5.70	2.57	2.28	2.08
52	0.106	0.063	68.00	1.90	2.08	2.17	2.08
59	0.352	0.169	63.80	4.00	2.63	2.25	2.08
56	1.415	0.816	59.20	2.40	3.13	2.37	2.08
47	0.098	0.060	58.10	1.90	2.08	2.19	2.08
55	0.126	0.067	55.10	2.60	2.18	2.25	2.08
53	0.694	0.416	53.60	2.10	2.96	2.38	2.08
54	0.644	0.399	52.70	2.10	3.01	2.24	2.08
48	1.893	1.201	50.80	1.90	3.29	2.47	2.08
50	0.217	0.132	50.70	1.90	2.47	2.40	2.08
49	665.899	461.930	48.70	1.30	6.40	2.41	2.08

**Fig. 9.4:**
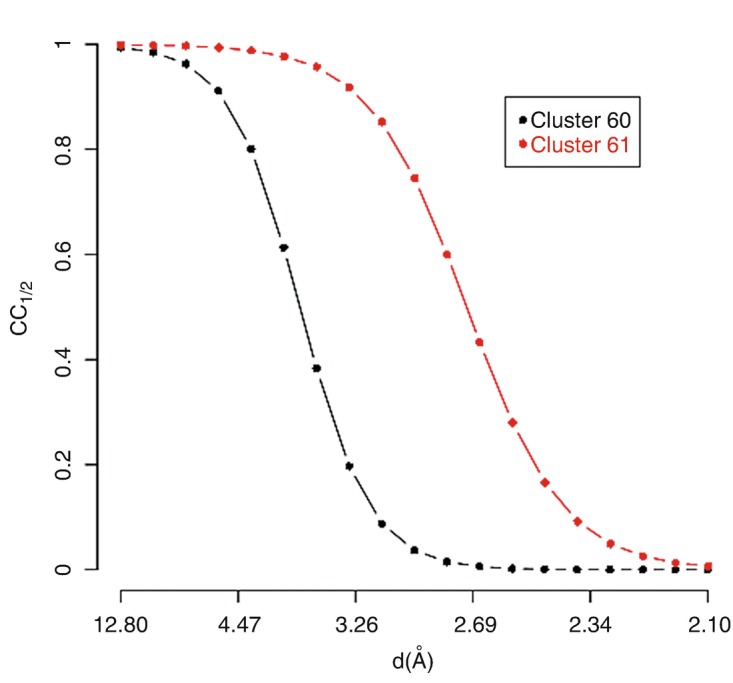
CC_1/2_ curves for the two datasets assembled with data from each of the two branches of the dendrogram for TehA

The two main branches of the dendrogram, cluster 60 and cluster 61, do not seem to provide good-quality data. This can be due to the resolution of 2.076 Å being too high and low data completeness (88.1 % for cluster 60 and 69.8 % for cluster 61). Furthermore, some of the individual datasets composing the clusters could have an inherent bad quality and, thus, needed to be filtered out. Filtering will be dealt with in the next section on combination and graphics modes. Here we will look at ways to improve statistics and completeness with respect to resolution. Plots of CC_1/2_ versus resolution (Evans 2006) for clusters 60 and 61 are displayed in Fig. 9.4.

Resolution seems better for cluster 61. This, though, can change after specific datasets have been filtered out. The overall CC_1/2_ seems to suggest that scaling at a resolution around 2.5 Å should work sensibly. A new execution of BLEND in *synthesis* mode, this time at resolution 2.5 Å, is reflected in the statistics summary in Table 9.4.

**Table 9.4:** Statistics after execution of BLEND in *synthesis* mode for the most complete merged datasets of TehA at 2.50 Å resolution

Cluster number	R_meas_	R_pim_	Completeness	Multi-plicity	Resolution CC_1/2_	Resolution Mn(I/sd)	Resolution Max
62	0.586	0.157	93.60	10.90	2.56	2.50	2.50
60	1.535	0.598	93.20	5.50	3.21	2.50	2.50
58	0.154	0.061	88.20	4.90	2.50	2.50	2.50
57	0.086	0.042	87.60	3.40	2.50	2.50	2.50
52	0.087	0.051	78.90	2.00	2.50	2.50	2.50
61	0.514	0.185	70.90	7.10	2.75	2.50	2.50
47	0.083	0.050	66.00	2.10	2.50	2.50	2.50
59	0.228	0.098	65.90	4.90	2.68	2.50	2.50
56	0.977	0.531	64.80	2.70	3.15	2.50	2.50
55	0.103	0.053	60.90	3.00	2.50	2.50	2.50
49	31.153	21.159	59.70	1.30	6.30	2.50	2.50
54	0.325	0.184	59.30	2.30	3.01	2.50	2.50
53	0.394	0.220	59.20	2.40	2.94	2.50	2.50
40	0.089	0.060	58.60	1.30	2.50	2.50	2.50
48	1.194	0.726	57.30	2.10	3.30	2.50	2.50

### H1R

The low isomorphism of many of the clusters is measured by the relatively high values of the aLCV parameter. For the H1R structure, we were trying to achieve good resolution, let us say at around 3 Å, hence it is appropriate to merge datasets with an aLCV value smaller than 3–4 Å. Resolution during scaling can also be fixed at 3 Å so that comparison among the various merged datasets becomes easier. In addition, resolution can still be extended in later runs of the program. The “bkeys.dat” keywords file used for this BLEND job includes the following lines:

RESOLUTION HIGH 3.0

TOLERANCE 100

The second keyword (“TOLERANCE”) has been added to stop *POINTLESS* from halting execution. When cell parameters are very different *POINTLESS* halts execution because it suspects crystals might come from different structures. Its tolerance has a default value of 2 and larger numbers increase this tolerance. “TOLERANCE 100” basically tells *POINTLESS* not to halt, even though crystals are very different. The BLEND run for this specific group of datasets in *synthesis* mode is started with the following command line:

blend -saLCV 3 < bkeys.dat

All files and statistics connected to this BLEND run are found in the directory “merged_files”. In this study, the three clusters with largest acceptable values of aLCV (less than 3) are clusters 13, 14 and 15. Although complete, these clusters have alarming merging statistics values and more work is needed to improve results. This is achieved by using the *combination* and *graphics* modes in BLEND as described in see Sect. 9.7.

## Combination and Graphics Modes (−c,−g): Improving Results from Synthesis Using Combination, Filtering and Graphics

Results obtained from running BLEND in *synthesis* mode, as said before, can be quickly surveyed by looking at the MERGING_STATISTICS. info file. Logs from all *POINTLESS* and AIMLESS jobs associated with each scaling and merging are also saved in the “merged_files” directory and therefore accessible if specific details are needed. If some of the new datasets show sufficient completeness, resolution and satisfactory data quality as described by R_meas_ and R_pim_, then the associated scaled and merged MTZ files in the same directory can be used for phasing and model building. However, in those cases where the above criteria are not met, it will be necessary to create new datasets that are not represented by the dendrogram nodes using *BLEND* in *combination* mode. At this stage it is also of great help to visualise dendrogram’s connections and associated merging statistics through *BLEND graphics* mode. It is practical to have completeness, R_meas_ and CC_1/2_ resolution visually associated with each node of the dendrogram in order to easily be able to judge the goodness of specific dataset combinations. Yet, the resulted annotated dendrogram would though appear cluttered with numbers, most of which likely not to be readable. For this reason, it has been found convenient in *BLEND* to introduce a *graphics* mode producing only parts of the annotated dendrogram focusing on specific clusters. Graphics files in PNG and PS format are produced and stored under a directory called “graphics” every time a run in *graphics* mode is executed. In the command line, the only required fields are the specific cluster number and the number of levels from the specified cluster that the user would like to visualise. The higher the number of clusters the more packed the annotated dendrogram will appear.

The other mode described in this section, the *combination* mode, is needed for all groupings not present in the dendrogram. Although the grouping suggested by BLEND, using cell parameters, tends to provide optimal datasets in terms of isomorphism and merging statistics, still there are many factors (quality of individual datasets, insufficient coverage of the reciprocal space, etc.) that preclude such grouping to be the best possible (Foadi et al. 2013). Therefore, it is ideal for the user to be allowed to try different dataset combinations, alternative to the ones represented by the clusters. This is the main reason why the *combination* mode was created in *BLEND*. All files and statistics produced by *BLEND* in *combination* mode are sequentially stored in a directory called “combined_files”.

### TehA

**Fig. 9.5:**
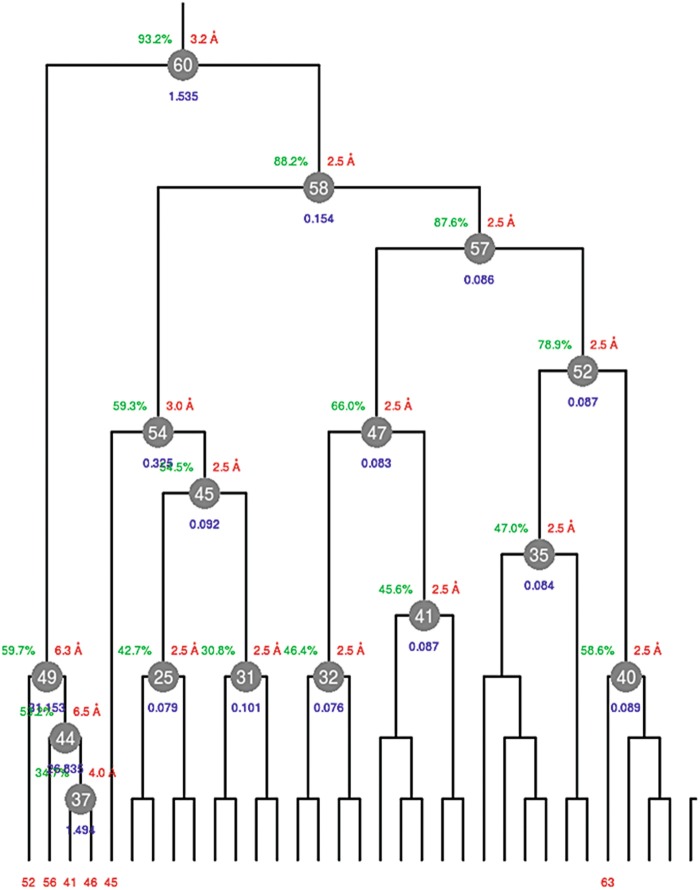
Annotated dendrogram created by BLEND in graphics mode around cluster 60, with 5 levels of annotation (“blend −g D 60 5”)

In the TehA data, cluster 60 is the only one with reasonable completeness, however its quality, at least in terms of resolution and R_meas_, is not great. One could ask if there are rogue individual datasets, part of cluster 60, responsible for the bad statistics. This can be easily investigated by running BLEND in *graphics* mode, focusing on cluster 60, and demanding sufficiently high number of cluster levels. The syntax command do this is

blend -g D 60 5

where (“−g”) means execution in *graphics* mode of the annotated dendrogram type focusing on cluster 60 to 5 levels of merging. The letter “D” in the syntax command at present is a default letter (it is envisaged that other types of plots will be added to future versions of BLEND. For these other types the “−g” of the graphics mode will be followed by letters different from “D”). The resulting annotated dendrogram is displayed in Fig. 9.5.

It is reasonably clear from Fig. 9.5 that cluster 49 (the small branch on the left) is the main responsible for the deterioration of data quality in cluster 60. Cluster 49 was composed of datasets 41, 46, 52 and 56.

To check if any of these datasets causes data quality to deteriorate, BLEND was executed in *combination* mode, starting from cluster 49 and subtracting one dataset at a time, using a special syntax developed for this purpose (see *BLEND* documentation at http://www.ccp4.ac.uk/html/blend.html)

blend -c [49] [[41]] < bkeys.datblend -c [49] [[46]] < bkeys.datblend -c [49] [[52]] < bkeys.datblend -c [49] [[56]] < bkeys.dat

Results from these 4 runs are shown in the first 4 lines of Table 9.5.

**Table 9.5:** Overall statistics obtained running BLEND in combination mode for the TehA case

Datasets	R_meas_	R_pim_	Completeness	Multi-plicity	Resolution CC_1/2_	Resolution Mn(I/sd)	Resolution Max
46,52,56	107.055	72.605	47.40	1.30	11.59	2.71	2.50
41,52,56	0.112	0.077	49.10	1.20	2.50	2.50	2.50
41,46,56	26.835	18.425	53.20	1.20	6.47	2.50	2.50
41,46,52	12.127	8.429	42.40	1.20	8.57	2.50	2.50
A	0.093	0.037	93.20	5.10	2.50	2.50	2.50
B	0.113	0.047	91.70	4.60	2.20	2.20	2.20

*A* cluster 60 without datasets 45 and 46 with resolution limited to 2.50 Å, *B* same as A with resolution extended to 2.20 Å

From the data shown in Table 9.5, it is clear that dataset 46 is a rogue dataset. Furthermore, it is also clear from the annotated dendrogram in Fig. 9.5 that dataset 45 also deteriorates statistics quality. Although cluster 45 has a reasonable R_meas_ value of 0.092 it jumps to a high value of 0.325 when the addition of dataset 45 turning cluster 45 into cluster 54. Thus, statistics were recalculated for cluster 60 without datasets 45 and 46 by the following command

blend -c [60] [[45,46]]

< bkeys.dat

Results from this run are shown in the fifth row of Table 9.5. The improvement observed is indisputable. It also was observed that without datasets 45 and 46 the resolution could be extended to 2.2 Å (row 6 in Table 9.5). Electron density maps obtained using the data just described, limited to 2.3 Å for comparison with data from a single cryo-cooled crystal, are shown in Axford et al. (2015).

### H1R

**Fig. 9.6:**
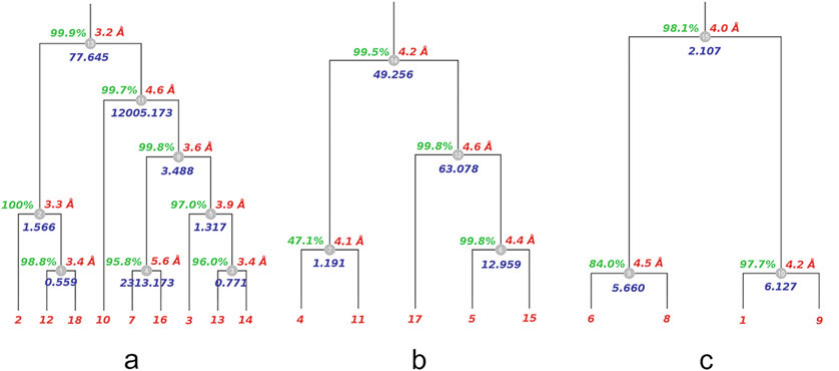
Annotated trees with statistics for clusters 13 (**a**), 14 (**b**) and 15 (**c**) obtained running BLEND*in synthesis* mode for H1R data

Three useful and visual summaries from statistics produced by BLEND in *synthesis* mode (see Sect. 9.6) were obtained in *graphics* mode with the following three command lines,

blend -g D 13 10

blend -g D 14 10

blend -g D 15 10

where the three clusters selected (13, 14, and 15) were the ones with acceptable largest values of the aLCV parameter, as explained in Sect. 9.6. The three plots produced are shown in Fig. 9.6 (see BLEND documentation for details of executions in graphics mode – http://www.ccp4.ac.uk/html/blend.html).

None of the statistics were satisfactory, but some of the estimated resolution values along with the CC_1/2_ parameter were interestingly high and certainly could be improved. The low values of aLCV and the relatively good resolution for many of the groups contrasts with the bad values of the merging statistics (R_meas_, R_pim_). This is normally due to situations where full convergence in the scaling process has not been reached. In the H1R study, this was mainly caused by the poor diffracting quality of the images or Radiation damage present in the datasets. With help from the annotated trees displayed in Fig. 9.6, and running BLEND in *combination* mode, datasets filtering could be tried in order to improve statistics, similarly to what was done for the TehA case study case. However, here a different approach was tried because of the bad quality data, mostly due to the effects of Radiation damage. Therefore, for each dataset forming a given cluster, images collected towards the end of the rotation sweep were eliminated so that R_meas_ and in particular R_pim_ values decreased while, completeness was not allowed to go below a given threshold. In *BLEND* this can be done automatically using a variant of the *combination* mode, “ −cP”, where “P” stands for “pruning”. In other words, during each cycle, specific fractions of images are eliminated from the dataset with the highest mean value of R_merge_, until target completeness (default is 95 %) or maximum number of cycles is reached. Pruning cycles are also halted if a whole dataset eventually turns out to be fully eliminated; this option can, in fact, be carried out differently with the simple combination mode, filtering out the specified dataset. Once cycling is completed, *BLEND* selects the final results for the cycle with lowest R_pim_. When the pruning variant of the *combination* mode is applied to clusters 13, 14 and 15, statistics generally seems to improve. Details are displayed in Table 9.6 where a comparison between the results from the *synthesis* mode and *combination* mode are shown.

**Table 9.6:** Statistics for clusters 13, 14, 15 in the H1R case before and after the elimination of the images with the pruning variant of the *combination* mode in BLEND (“−cP”)

		R_meas_		R_pim_		Resolution (CC1/2 > 0.3)		Completeness (%)		Multiplicity	
Clusters	Images pruned	Before	After	Before	After	Before	After	Before	After	Before	After
13	404	77.645	0.729	13.153	0.146	3.2	3.0	99.9	99.9	31.6	22.4
14	186	49.256	1.819	16.933	0.598	4.2	3.9	99.5	98.3	11.8	9.0
15	0	2.107	2.107	0.675	0.675	4.0	4.0	98.1	98.1	8.7	8.7

The procedure automatically selects the cycle with the best R_pim_ Improvements were evident for clusters 13 and 14. However, for cluster 15, any pruning cycle has returned data with worst statistics therefore results are unchanged in the table for this cluster

**Fig. 9.7:**
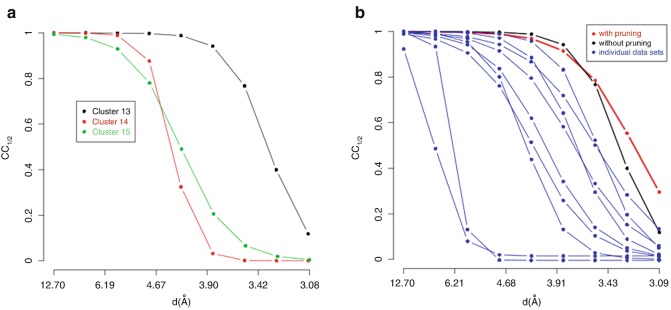
Beneficial effects of multiple-crystals datasets on resolution, as quantified by CC_1/2_ curves. (**a**) Resolution for cluster 13 is far better than resolution for clusters 14 and 15. (**b**) Merging data from multiple isomorphous crystals helps to extend resolution. Each *blue curve* describes the CC_1/2_ value for all individual datasets composing cluster 13 (*black curve*). Resolution is further extended when parts of data affected by Radiation damage are eliminated using the pruning variant of BLEND*combination* mode (*red curve*)

Perhaps the most interesting result concerning the processing of these data is an increase of the final resolution, as illustrated by the improved CC_1/2_ curves (see Fig. 9.7 for details).

## Conclusions

In this chapter it has been shown how BLEND can be used effectively to analyse and manage X-ray diffraction data from Multiple crystals of membrane proteins. Its three main modes (*analysis, synthesis and combination*), together with specific variants, enable users to explore data isomorphism and data quality, and provide a flexible and easy-to-use platform to create complete datasets to be used in the follow up stages of phasing, model building and refinement.

BLEND Cluster analysis, based on unit cell differences, is particularly useful for membrane protein data analysis, because of the high-content solvent and detergent-aided packing, making this class of proteins particularly susceptible to large cell size variations. A quantitative and practical measure of such variability is provided by LCV and aLCV. Groups of data with small values of these quantities can normally be merged into more complete and redundant data, using *synthesis* and *combination* modes. In particular, unwanted datasets can be filtered out of specific groups using the *combination* mode.

One last but important comment, concerns BLEND’s flexibility as represented by its propensity to adapt to both easy and difficult data. While the software can produce complete datasets with minimal user’s intervention when data have inherently good quality, a more interactive use of the software can lead to complete datasets in those cases where traditional data merging produce unacceptable or unusable data.
